# Structure analysis of supported disordered molybdenum oxides using pair distribution function analysis and automated cluster modelling

**DOI:** 10.1107/S1600576719016832

**Published:** 2020-02-01

**Authors:** Troels Lindahl Christiansen, Emil T. S. Kjær, Anton Kovyakh, Morten L. Röderen, Martin Høj, Tom Vosch, Kirsten M. Ø. Jensen

**Affiliations:** a Department of Chemistry and Nanoscience Center, University of Copenhagen, Copenhagen, DK-2100, Denmark; b Niels Bohr Institute and Nanoscience Center, University of Copenhagen, Copenhagen, DK-2100, Denmark; c Danish Technological Institute (DTI), Nano Production and Micro Analysis, Taastrup, 2630, Denmark; d Department of Chemical and Biochemical Engineering, Technical University of Denmark (DTU), Kongens Lyngby, DK-2800, Denmark

**Keywords:** molybdenum oxides, nanostructure, pair distribution function analysis, supported catalysts, automated modelling

## Abstract

Molybdenum oxides and sulfides on various low-cost high-surface-area supports are excellent catalysts for several industrially relevant reactions. The surface layer structure of these materials is, however, difficult to characterize due to small and disordered MoO*_x_* domains. Here, it is shown how X-ray total scattering can be applied to gain insights into the structure through differential pair distribution function (d-PDF) analysis, where the scattering signal from the support material is subtracted to obtain structural information on the supported structure. MoO_*x*_ catalysts supported on alumina nanoparticles and on zeolites are investigated, and it is shown that the structure of the hydrated molybdenum oxide layer is closely related to that of disordered and polydisperse polyoxometalates. By analysing the PDFs with a large number of automatically generated cluster structures, which are constructed in an iterative manner from known polyoxometalate clusters, information is derived on the structural motifs in supported MoO_*x*_.

## Abstract 2

Using pair distribution function analysis, the structure of amorphous supported molybdenum oxides is characterized using a new automated data modelling approach.

## 1. Introduction   

Nanostructured metal oxides of, for example, molybdenum, chromium or vanadium supported on cheap high-surface-area supports, *e.g.* titania, alumina or zeolites, are attractive candidates in many areas of heterogeneous catalysis (Zaera, 2013 ▸; Shiju & Guliants, 2009 ▸; Munnik *et al.*, 2015 ▸; Banares, 1999 ▸; Zhang *et al.*, 2013 ▸). To develop the field further and realize the full potential of these nanostructured oxides, a comprehensive understanding of the structure/property relationship is essential (Macht & Iglesia, 2008 ▸; Bell, 2003 ▸). Currently, structural information for these nanomaterials is obtained through spectroscopic methods, *e.g.* Raman spectroscopy, X-ray photoelectron spectroscopy, UV–visible or X-ray absorption, as the small domain sizes and amorphous nature of the materials challenge traditional microscopy and diffraction techniques (Macht & Iglesia, 2008 ▸). X-ray total scattering, where diffuse scattering arising from disordered atomic arrangements is included in the data treatment, has over the past decade evolved into a powerful technique for structural characterization of nanomaterials with limited structural order. As opposed to traditional powder diffraction, total scattering allows for observation of the atomic arrangement in nanostructures without long-range order and with ångström resolution (Billinge & Kanatzidis, 2004 ▸).

**Figure 1:**
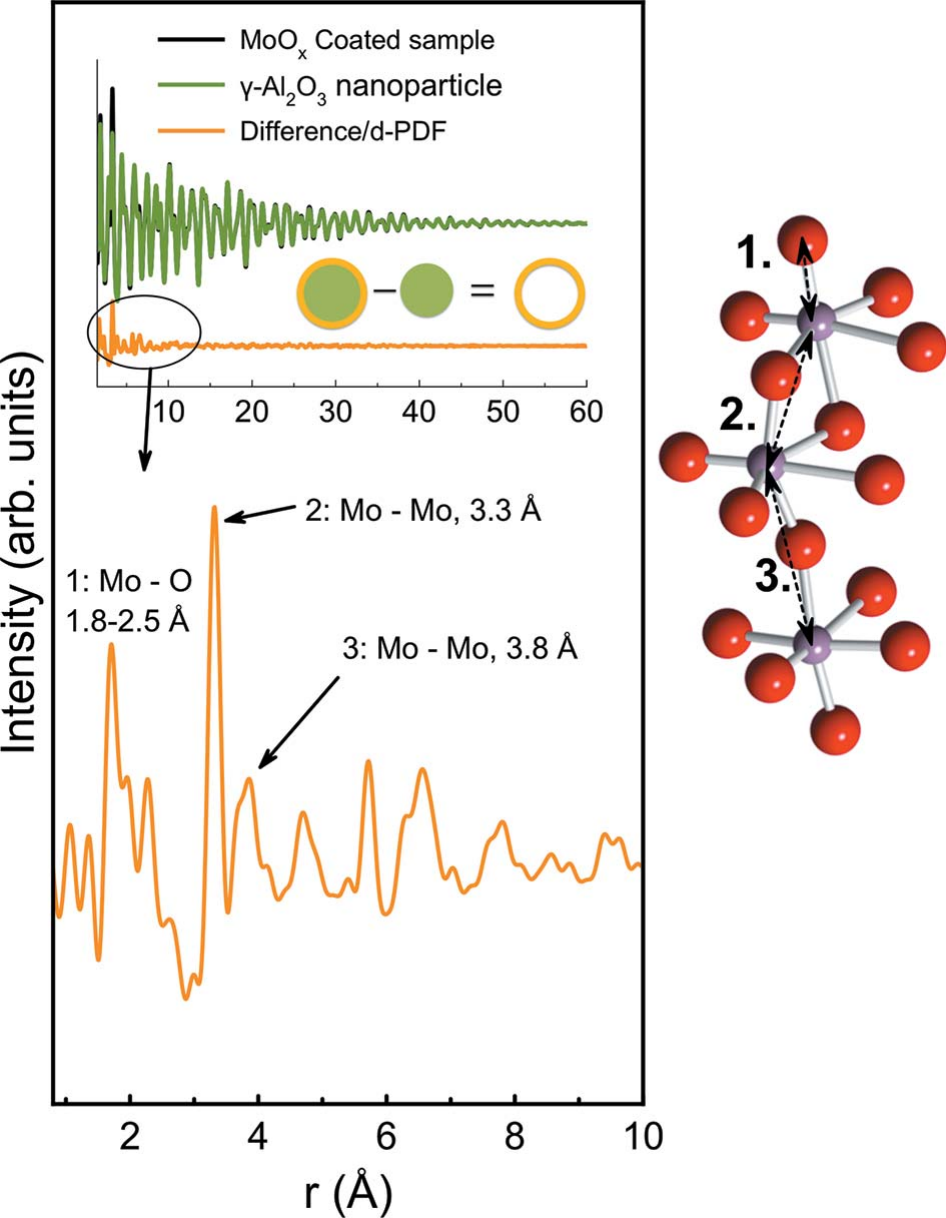
Illustration of the generation of the d-PDF. A PDF is acquired from the supported sample as well as from the clean support, and the d-PDF is obtained by subtraction of the former from the latter. This results in a PDF expressing the structure of the supported material only. This is illustrated here for molybdenum oxide supported on alumina nanoparticles. The most significant peaks of the d-PDF can be assigned, namely the Mo–O peak (1.8–2.5 Å), the edge-sharing Mo–Mo peak (3.3 Å) and the corner-sharing Mo–Mo peak (3.8 Å).

Total scattering data are usually analysed through the pair distribution function (PDF), which is the Fourier transform of the scattering signal; it expresses the probability of finding a pair of atoms at a distance *r*, which provides an intuitive way of analysing structures. PDF analysis has previously been shown to be a very powerful technique to elucidate atomic structures of supported materials, *e.g.* arsenate or large cations adsorbed on γ-Al_2_O_3_, ferrihydrite or δ-MnO_2_ (Harrington *et al.*, 2010 ▸; Li *et al.*, 2011 ▸; van Genuchten & Pena, 2016 ▸; Chupas *et al.*, 2011 ▸). By acquiring X-ray total scattering data and obtaining PDFs for both the clean support and the sample on the support, and subtracting the former from the latter, it is possible to obtain a PDF describing the supported structure only, as demonstrated in Fig. 1 ▸. The resulting PDF is often referred to as the differential PDF (d-PDF) (Chapman & Beauchamp, 2006 ▸).

Here, we present d-PDF studies of supported nano­structured MoO_*x*_ samples on different supports. We use a new method for analysing the obtained d-PDFs of disordered nanoclusters, where the data are fitted with a large number (thousands) of cluster models, which are automatically built on the basis of known metal oxido cluster structures. A similar approach to ‘automated modelling’ has recently been successfully applied to structural analysis of metal clusters (Banerjee *et al.*, 2019 ▸). By studying metal oxido clusters and fitting this large number of structures to the d-PDF, we can extract information on the structural motifs present in the disordered molybdenum oxide systems. We first demonstrate this method for nanostructured MoO_*x*_ on γ-Al_2_O_3_. This system has received much attention due to its use in oxidative dehydrogenation of small alkanes and other industrially relevant catalytic reactions (Cavani *et al.*, 2007 ▸; Høj *et al.*, 2014 ▸; Setnička *et al.*, 2015 ▸). Subsequently, we apply the same method to MoO_*x*_ supported on zeolites. MoO_*x*_ supported on zeolites are promising candidates for catalysing the conversion of waste methane into liquid aromatic hydrocarbons (Gao *et al.*, 2015 ▸), but there is limited structural knowledge of the MoO_*x*_ layer (Li *et al.*, 2006 ▸). We show that the MoO_*x*_ layer on both alumina and zeolites under ambient conditions consists of [MoO_6_] octahedra forming small clusters with a structural domain length of approximately 1 nm. The structures, which are hydrated from atmospheric air, contain similar motifs to known polyoxometalate structures found in solution, although they are not monodisperse and include a much larger degree of structural disorder.

## 2. Results and discussion   

### 2.1. Molybdenum oxide supported on γ-Al_2_O_3_   

Several hypotheses regarding the structure of molybdenum oxide on alumina have been presented in recent decades. For high MoO_*x*_ loadings, corresponding to more than monolayer MoO_*x*_ coverage on the support, crystalline MoO_3_ is formed on the particles, while a nanostructured and catalytically active structure forms at lower loadings (Mestl & Srinivasan, 1998 ▸). It has previously been speculated that the supported nano­structured molybdenum oxide has a local structure similar to that of MoO_3_. However, further structural work, using primarily Raman spectroscopy, has indicated that at low loadings (below monolayer coverage) isolated, tetrahedrally coordinated [MoO_4_] units occupy the alumina surface and bind directly to the alumina structure (Chen *et al.*, 2001 ▸; Drake & Stair, 2017 ▸; Tsilomelekis & Boghosian, 2013 ▸; Wachs, 1996 ▸). When the MoO_*x*_ coverage is large enough for monolayer coverage, the dominant structural unit is believed to be octahedral [MoO_6_], and it has been suggested to be in conformations similar to polymolybdate units found in solutions such as [Mo_7_O_24_]^6−^ or [Mo_8_O_26_]^4−^ in oligomeric chains (Mestl & Srinivasan, 1998 ▸). The structural arrangements are highly dependent on several factors, including the support and the degree of hydration of the samples from atmospheric air (Deo & Wachs, 1991 ▸). No unambiguous surface structure model exists with regards to molybdate species and the extent of ordering (Tsilomelekis & Boghosian, 2013 ▸). Using d-PDF, we can test existing hypotheses and develop a structural model for the supported molybdenum oxide.

**Figure 2:**
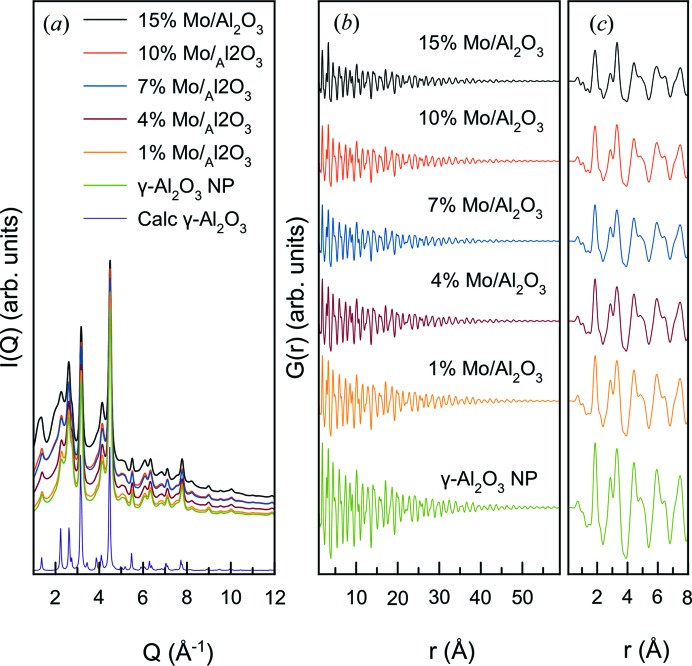
(*a*) X-ray total scattering data obtained from supported MoO_*x*_/γ-Al_2_O_3_ samples, as well as data showing the calculated signal from ordered γ-Al_2_O_3_ nanoparticles. (*b*) PDFs derived from the measured X-ray total scattering data. (*c*) Local region of the PDFs.

Fig. 2 ▸(*a*) shows X-ray total scattering data for a range of MoO_*x*_/γ-Al_2_O_3_ samples with molybdenum content from 0 to 15 wt% Mo. The samples all show very broad Bragg reflections, which can be assigned to the γ-Al_2_O_3_ support (Paglia *et al.*, 2003 ▸). No additional Bragg peaks are seen in any of the samples; however, the presence of increasing amounts of MoO_*x*_ is seen in the scattering pattern as a rise in diffuse scattering intensity over the entire *Q* range. Diffuse scattering originates from nanostructured, amorphous and highly disordered phases, and the increase in diffuse scattering is thus indicative of a disordered MoO_*x*_ layer on the substrate, agreeing with previous observations. Note that the disordered nature of the alumina support also contributes to the diffuse scattering signal.

**Figure 3:**
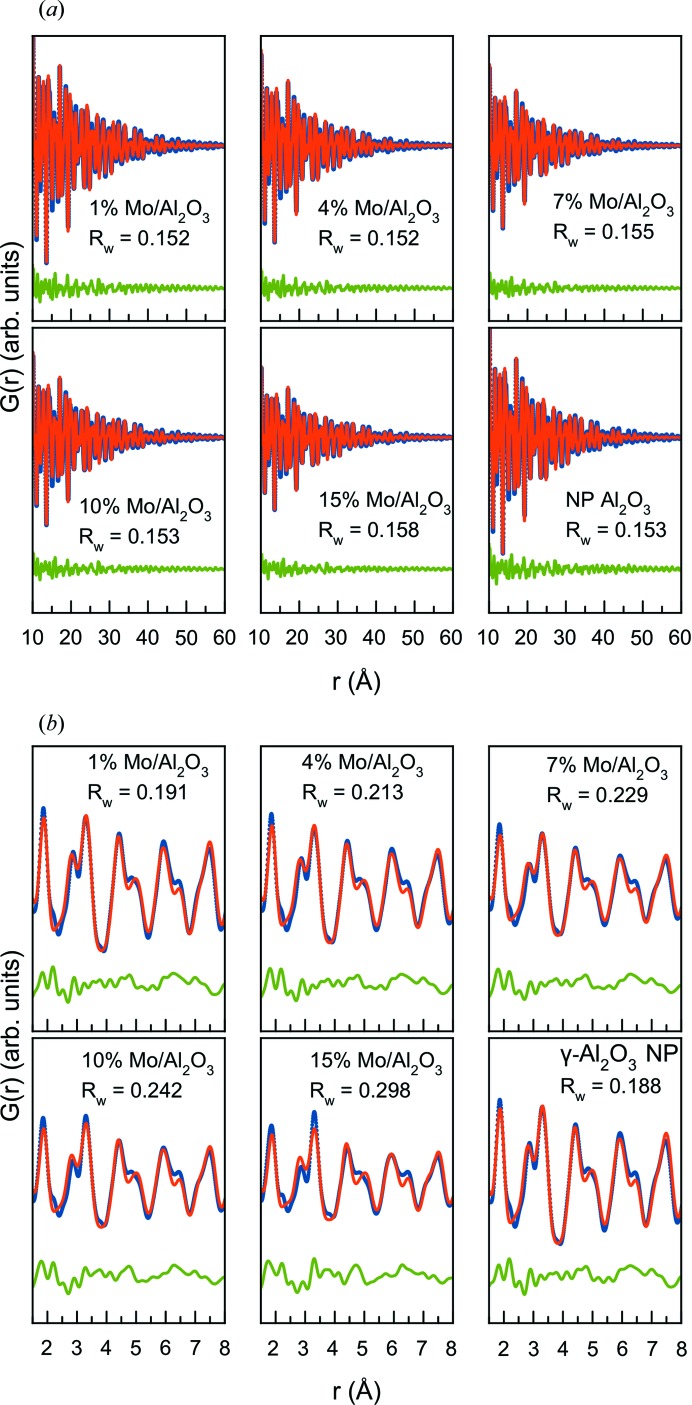
(*a*) Real-space fits of the measured PDFs in long range (10–60 Å) using the bulk tetragonal γ-Al_2_O_3_ structure and (*b*) fits to the local range (1–8 Å) using the fine-scale alumina model of Paglia *et al.* (2006 ▸). In all fits, the experimental PDF is displayed as blue dots, the calculated fit as a red line and the difference curve as a green line.

Before calculating the d-PDFs from the X-ray total scattering data for further structural analysis, the PDFs from all samples are analysed to establish the existence of a nano­structured MoO_*x*_ layer and to facilitate calculation of the d-PDFs. The PDFs calculated from the data sets in Fig. 2 ▸(*a*) are shown in Fig. 2 ▸(*b*). All PDFs show similar features, except in the local range [*ca* 1–8 Å, Fig. 2 ▸(*c*)]. The PDFs show damping at high *r*, and when analysed in the range 10–60 Å the data can be fitted by the tetragonal γ-Al_2_O_3_ model (space group *I*4_1_/*amd)* (Paglia *et al.*, 2003 ▸) using a damping factor for spherical particles (sp-diameter) after taking into account instrumental damping. The refinements can be seen in Fig. 3 ▸(*a*) (10–60 Å range) and yield unit-cell parameters of *a*, *b* ≃ 5.62 Å and *c* ≃ 7.83 Å and an sp-diameter of *ca* 8 nm when assuming monodisperse spherical particles. The agreement of the fitted parameters and *R*_w_ values between the six samples demonstrates that the structure of the γ-Al_2_O_3_ support is largely unaffected by the MoO_*x*_ coating. All refinement results are given in Table S1 in the supporting information.

**Figure 4:**
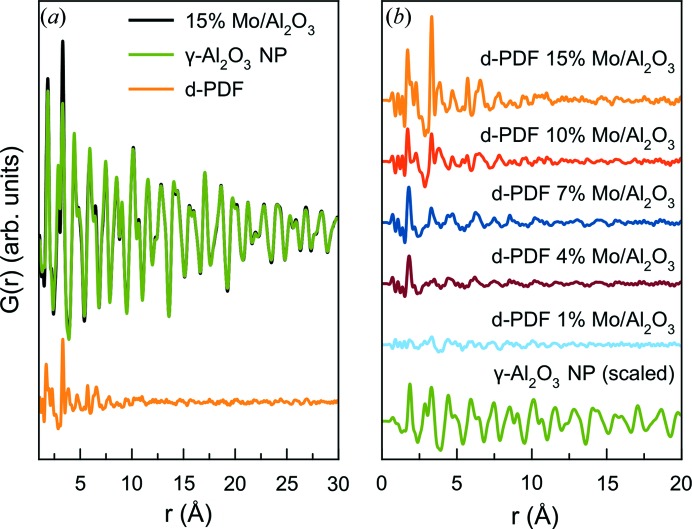
Generation of d-PDFs. (*a*) Scaling and subtraction of the NP γ-Al_2_O_3_ PDF from the 15% Mo/γ-Al_2_O_3_ PDF. (*b*) Comparison of generated d-PDFs.

The bulk γ-Al_2_O_3_ model does not fit the local structure of the data, no matter what the MoO_*x*_ loading. This is well known for γ-Al_2_O_3_ and has previously been studied in detail by Paglia *et al.* (2006 ▸), who derived a local structure model for the compound. A fit using this local structure model is shown in Fig. 3 ▸(*b*) (1–8 Å range). The model shows a good fit to the data from pure γ-Al_2_O_3_ nanoparticles (NPs) and the 1% Mo/γ-Al_2_O_3_ sample, which is comparable in fit quality to those reported previously (Paglia *et al.*, 2006 ▸). As expected, an increasing discrepancy in the fit and *R*_w_ factor can be observed for increasing MoO_*x*_ loading. Importantly, this increase is only observed in the local-range fit, meaning that the MoO_*x*_ only shows short-range structural correlations. This facilitates the generation of the d-PDFs, as the intensity of the peaks originating from the support seen in the long-range region can be scaled to match in intensity with the MoO_*x*_/γ-Al_2_O_3_ sample before subtraction. The scaling and generation of the d-PDF are demonstrated for the sample with 15% Mo in Fig. 4 ▸(*a*), where the substrate PDF has been scaled to match the intensity in the sample PDF for a γ-Al_2_O_3_ peak at 17.3 Å. In Fig. S1 we demonstrate that it is possible to obtain equivalent PDFs by subtracting the scattering signal from the substrate in *Q* space before the Fourier transform; however, subtraction in *r* space was preferred due to the convenient and reliable method of scaling the substrate intensity before subtraction.

All d-PDFs are shown in Fig. 4 ▸(*b*) where they are compared with the clean γ-Al_2_O_3_ NP sample. The PDF from the sample containing 1% Mo shows no PDF peaks that can confidently be assigned to MoO_*x*_ structural motifs. This is surprising as the d-PDF technique has previously been shown to be sensitive to low loading percentages using elements with lower scattering power than Mo (Chapman *et al.*, 2006 ▸; Harrington *et al.*, 2010 ▸). However, this observation may be explained by the nature of the highly defective Al_2_O_3_ structure (Paglia *et al.*, 2003 ▸), where Mo may be incorporated into the γ-Al_2_O_3_ structure by occupying cation vacancies in the surface. If this is the case, the [MoO_*x*_] signal is then mostly subtracted along with the γ-Al_2_O_3_ support.

Distinct structural peaks are seen in d-PDFs for samples with MoO_*x*_ loading higher than 4% Mo. The first PDF peak from the MoO_*x*_ structure is at 1.8 Å, which falls within the typical Mo—O bond distances (Hardcastle & Wachs, 1990 ▸; Pope, 1991 ▸), confirming the presence of [MoO_*x*_] units on the surface. On the basis of known crystal structures, the Mo–Mo distances are expected to be 3.3 Å (edge-sharing [MoO_6_] octahedra) or 3.8 Å (corner-sharing [MoO_6_] octahedra or tetrahedra), as illustrated in Fig. 1 ▸. In the sample with 4% Mo loading, no clear peaks are seen in this range, indicating that the [MoO_*x*_] units are isolated on the substrate surfaces; this is in agreement with observations in other studies of low-loading MoO_*x*_/γ-Al_2_O_3_ samples, suggesting the presence of tetrahedral monomolybdate ions, [MoO_4_]^−^, in hydrated samples (Mestl & Srinivasan, 1998 ▸; Drake & Stair, 2017 ▸). In the sample with 7% Mo, increased intensity is seen in the PDF at *ca* 3.3 Å and at higher *r* values, which shows the beginning of the formation of larger MoO_*x*_ cluster structures. These peaks are clearly present in the samples with 10–15% Mo loading, where PDF peaks to *ca* 12 Å are apparent, which is also the wt% where monolayer coverage is expected to form (Høj *et al.*, 2014 ▸). These two samples show distinct peaks at both 3.3 and 3.8 Å, indicating that both edge- and corner-sharing [MoO_*x*_] motifs are present in the clusters. A peak is found at *ca* 6.6 Å, *i.e.* double the edge-sharing distance of 3.3 Å, which indicates that edge-sharing [MoO_*x*_] units are a dominant structural motif. The d-PDFs thus clearly show that, in these samples, the [MoO_*x*_] units are not isolated but form a nanostructure with correlation between several units which must be sharing predominantly edges, but also to some degree corners. This is similar to polymolybdate ions primarily built from [MoO_6_] octahedra in solution (Pope & Müller, 1991 ▸). Such structures are expected for supported oxides under ambient conditions, as moisture from the air adsorbs on the material surface leading to solvation of the oxide species (Bañares & Wachs, 2002 ▸).

Note that, in our analysis, we have not considered peaks originating from Al–Mo correlations, *i.e.* between the substrate and MoO_*x*_. An Mo–Mo correlation will yield approximately four times the intensity of an Mo–Al correlation in the PDF due to the larger scattering power of Mo. The Al–Mo correlation should be most clear for the [MoO_4_] tetrahedra in the 4% sample, where an extended network of Mo–Mo correlation has yet to form. However, as mentioned, very little signal is seen in the 4% d-PDF other than the Mo–O peak. The lack of Mo–Al correlation can possibly be explained by the low scattering power of Al and large variations in angle/bond lengths of the Al—O—Mo unit on the disordered surface of the particles.

**Figure 5:**
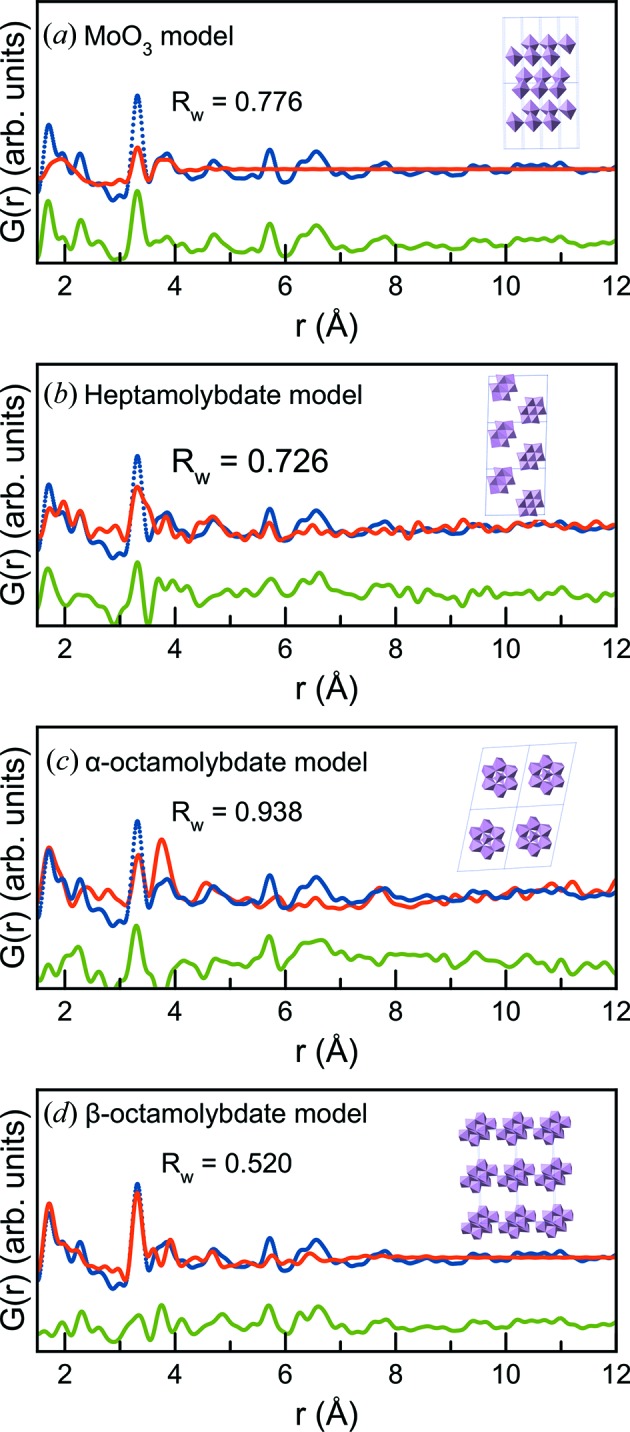
PDF fits to the data obtained from 15% Mo/γ-Al_2_O_3_ data with well-known crystal structures: (*a*) MoO_3_, (*b*) heptamolybdate (NH_4_)_6_Mo_7_O_24_(H_2_O)_4_, (*c*) α-octamolybdate (C_18_H_20_N_4_)_2_[Mo_8_O_26_] and (*d*) β-octamolybdate (NH_4_)_6_(Mo_8_O_26_)(H_2_O)_4_. In all fits, the experimental PDF is displayed as blue dots, the calculated model as a red line and the difference curve as a green line.

For further analysis of the larger cluster structure, we focus on the sample containing 15% Mo. We first attempted to fit the d-PDFs with existing crystalline models which have previously been suggested for supported molybdenum oxides. The lack of long-range order was taken into account with a spherical damping function (Gilbert, 2008 ▸), reducing the intensity of PDF peaks in the high-*r* region after accounting for instrumental PDF damping. This analysis was done using *PDFgui* (Farrow *et al.*, 2007 ▸). Four fits are displayed in Fig. 5 ▸ using models of structures most commonly discussed in relation to supported molybdenum oxide, *i.e.* MoO_3_, [Mo_7_O_24_]^6−^ (hepta­molybdate), and [Mo_8_O_26_]^4−^ (octamolybdate) in both the α and β isomers (Bridgeman, 2002 ▸). In the MoO_3_ model, the structure in space group *Pbnm* was used, while [Mo_7_O_24_]^6−^ was derived from (NH_4_)_6_Mo_7_O_24_(H_2_O)_4_ (Evans *et al.*, 1975 ▸), and the α and β [Mo_8_O_26_]^4−^ models from (C_18_H_20_N_4_)_2_[Mo_8_O_26_] (Wang *et al.*, 2007 ▸) and (NH_4_)_6_(Mo_8_O_27_)(H_2_O)_4_ (Böschen *et al.*, 1974 ▸), respectively. The models were prepared by removing all atoms except Mo atoms and O atoms bonded to Mo from the unit cell. The unit-cell parameters, isotropic atomic displacement parameters, a parameter relating to the correlated atomic motion (delta2) (Jeong *et al.*, 1999 ▸) and a parameter for the spherical damping were refined along with a scale factor. Fit results are listed in Table S2.

The fit to the MoO_3_ model, shown in Fig. 5 ▸(*a*), fails to fit anything but the closest edge- and corner-shared peaks. The [Mo_7_O_24_] model [Fig. 5 ▸(*b*)] performs slightly better and provides the correct peak position for some higher-order peaks, thus giving a better description of the connection between the [MoO_*x*_] units. However, the model contains too much disorder in the edge-sharing octahedra, making the peak at 3.3 Å too broad, and the intensity in the peaks above 5 Å is poorly described. The α-[Mo_8_O_26_] model in Fig. 5 ▸(*c*) overestimates the 3.8 Å peak compared with the 3.3 Å peak and also fails to provide a good description for peaks over 5 Å. The β-[Mo_8_O_26_] structure provides the best fit of the three models as seen in Fig. 5 ▸(*d*), although the peak intensities above 5 Å are still poorly described.

When fitting crystal structures to the PDF, as is done here, interactions between the individual clusters are also part of the calculated PDF. While heptamolybdate and the two octamolybdate isomers have similar local structure motifs in the clusters, the relations between the individual cluster units in the crystal structures vary significantly. In (NH_4_)_6_Mo_7_O_24_(H_2_O)_4_ and (C_18_H_20_N_4_)_2_[Mo_8_O_26_] the [Mo_7_O_24_] and α-[Mo_8_O_26_] units are isolated with a distance of 5–7 Å between the units, while in (NH_4_)_6_(Mo_8_O_26_)(H_2_O)_4_ the β-[Mo_8_O_26_] units form polymeric chains by corner sharing between the units, as seen in the inset of Fig. 5 ▸(*d*). While the clusters present on the substrate surface may be arranged in a semi-ordered manner, there is no reason to believe that this is similar to the arrangement of the clusters in the crystal structures. To simplify the models, we therefore attempt to treat the clusters as finite structural models, without assuming periodicity or symmetry. This is done by using the Debye scattering equations to calculate the PDFs (Gelisio & Scardi, 2016 ▸) from atomic coordinates applying the *Diffpy-CMI* program package (Juhás *et al.*, 2015 ▸). The *xyz* coordinates of the cluster models used can be found online (Lindahl Christiansen *et al.*, 2019 ▸).

**Figure 6:**
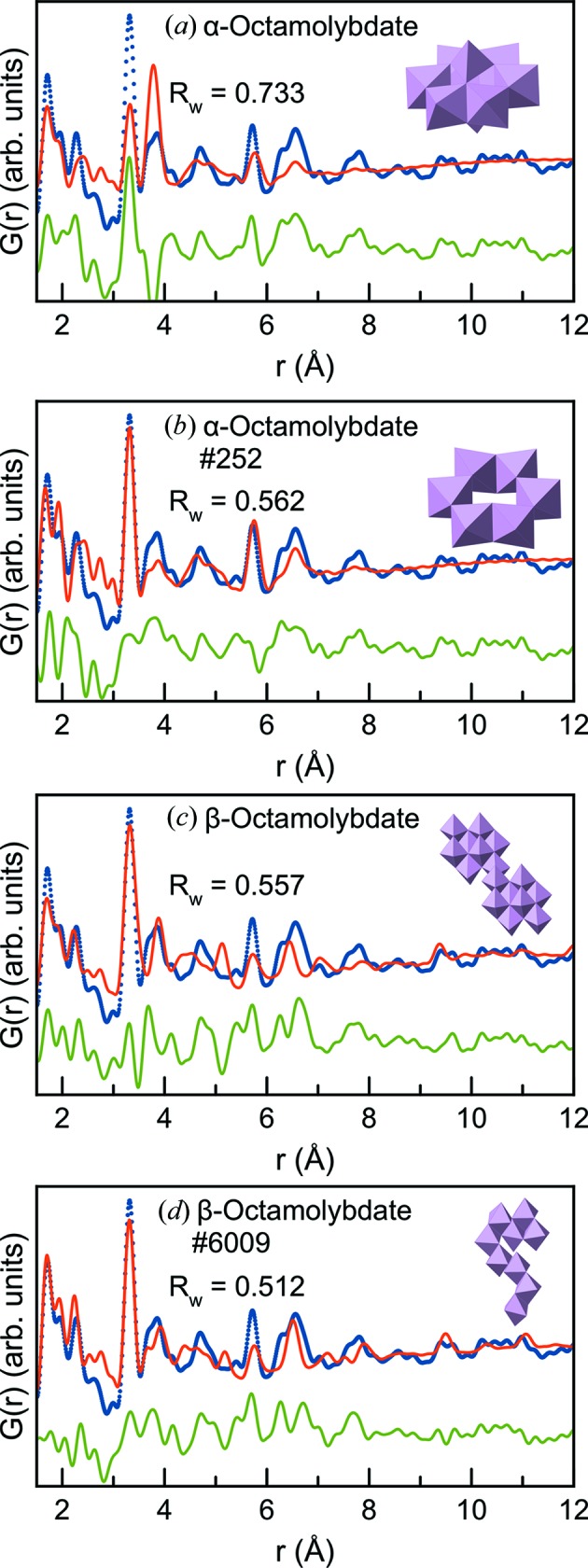
(*a*) Fit of the α-[Mo_8_O_26_] cluster structure to the d-PDF obtained from the sample with 15% Mo. (*b*) Fit of the best-fitting α-[Mo_8_O_26_]-derived cluster structure. (*c*) Fit of the β-[Mo_8_O_26_] cluster structure to the d-PDF. (*d*) Fit of the best-fitting β-[Mo_8_O_26_]-derived cluster structure. In all fits, the experimental PDF is displayed as blue dots, the calculated model as a red line and the difference curve as a green line.

Debye fits using the α-[Mo_8_O_26_] cluster and two units of the β-[Mo_8_O_26_] extracted from the crystal structures used for the pseudo-crystalline models are shown in Figs. 6 ▸(*a*) and 6 ▸(*c*), respectively. The fits were performed in a highly constrained manner to avoid unphysical bond distances and geometries, allowing only expansion or contraction of the full cluster structure and otherwise only fitting isotropic atomic dis­place­ment parameters of the Mo and O atoms (Jensen *et al.*, 2016 ▸). The fit quality is comparable to the pseudo-crystalline fits seen in Figs. 5 ▸(*c*) and 5 ▸(*d*), although the fit above 5 Å is improved for β-[Mo_8_O_26_] as the envelope function now does not quench the intensity at higher *r* values. As seen in Table S3, the anisotropic expansion/contraction parameters are close to 1 (0.98–1.00), illustrating that the geometry and structure of the cluster are kept in the fitting process. The fits with the cluster models and the damped crystalline models can both be greatly improved by refining the Mo atomic positions in the structures; however, this introduces far too many parameters in the model compared with the information available in the PDF and results in unphysical atomic distances in the model.

While the fits above demonstrate that the octamolybdate clusters give a reasonable fit to the data, it is clear that the molybdenum oxide species present in the sample are not monodisperse units of either heptamolybdate or octamolybdate clusters as has previously been suggested (Mestl & Srinivasan, 1998 ▸). This is highlighted when considering other samples, where the clusters are in fact monodisperse, *i.e.* as is the case for molybdate ions and other polyoxometalates in aqueous solution (Juelsholt *et al.*, 2019 ▸). Examples of such structures and fits to data from polyoxometalate clusters are shown in Fig. S2. These fits show a much smaller discrepancy between fit and data, and the Mo–Mo peaks are better defined. If comparing this with the fits shown in Figs. 6 ▸(*a*) and 6 ▸(*c*), it is clear that the MoO_*x*_ surface layer is disordered and polydisperse, consisting of a range of different cluster sizes with a different number of [MoO_6_] octahedra. To demonstrate this, we generated a large number of new models by iteratively modifying known polyoxometalate cluster structures. New structures were created by iteratively removing [MoO_6_] octahedra from a starting cluster so that all possible sizes of a given Mo–oxido cluster are created. Using *e.g.* the octamolybdate cluster structures shown in Figs. 6 ▸(*a*) and 6 ▸(*c*) (insets) as starting models, one or more of the Mo atoms were removed, thus generating new clusters. Whenever an Mo atom was removed, all O atoms not bonded to another Mo were also removed from the structure. In this way, 2^8^ = 256 and 2^16^ = 65 536 new models were created from α- and β-[Mo_8_O_26_], respectively. The new structures will thus contain the same octahedra/tetrahedra as the octamolybdate clusters, but in different arrangements. We fitted all the new clusters to the d-PDF in the *r* range of 3–12 Å. This range was chosen so as to not let the broad Mo–O peak centred at 1.8 Å dominate the fit result, but rather focus on the Mo–Mo correlations. Note that, due to the symmetry of the clusters, some of the newly generated clusters will be duplicates. Duplicate structures are identified by checking if any other models yield the same *R*_w_ value and have the same number of Mo and O atoms in the model. For α-[Mo_8_O_26_] the number of new, unique models is 160, and for β-[Mo_8_O_26_] it is 14 054.

Several cluster models were fitted initially to determine good candidates for starting structures. A good candidate provides a reasonable fit, but most importantly features peaks in the correct positions, as the method fundamentally just changes relative intensity between peaks. The tested structures are listed in Table S4.

Figs. 6 ▸(*b*) and 6 ▸(*d*) show the two best fits obtained using the method starting from α-[Mo_8_O_26_] and β-[Mo_8_O_26_], respectively. From comparison with the original fits, it is evident that both the visual fit and *R*_w_ value are improved, specifically the 3.3 and 3.8 Å peaks are better fitted by the new model, as well as the peaks past *r* = 6 Å for the β-[Mo_8_O_26_]-derived models. However, the models still have significant discrepancies, which can be ascribed to the disordered and polydisperse nature of the nanostructure. While the structure presented here should not be seen as a single representation of the clusters present on the alumina surface, we can use our results to identify important structural motifs by considering the trends in the newly generated models that improve the fit. The results, discussed below, are summarized in Table S5.

Considering first the 160 models of α-[Mo_8_O_26_], 57 (36%) gave a better fit to the experimental PDF than the starting α-[Mo_8_O_26_] model, and 45 (28%) lower the *R*_w_ value by 5% or more. An overview of the fit quality (*R*_w_ values) for all cluster structures can be found in Fig. S3. The minimum *R*_w_ value is reached for models that contain around five to six Mo atoms; however, as the models do not describe the peaks above 7 Å, this is probably too few Mo atoms to provide a full description of the nanostructure. Comparing the structures of the initial model with the best-fitting model in the insets of Fig. 6 ▸(*a*) and Fig. 6 ▸(*b*), respectively, it can be seen that the improved model is identical to the initial model, except that the tetrahedral [MoO_4_] units present in the structure have been removed. Of the 160 newly generated models, 120 (75%) contain tetrahedral units; however, this number falls to 30 of 57 (53%) for the models improving the original fit, and 18 of 45 (40%) for the models improving the fit by 5% or more. Thus, it appears that the best models do not contain any tetrahedral units, and it is likely that they are not a dominant structural unit. This is supported by an identical fall in the prevalence of tetrahedral units when applying the same method using decamolybdate as a starting point (Table S6), another polyoxometalate which also contains tetrahedral [MoO_4_] units.

Evaluation of the 14 054 new structures for β-[Mo_8_O_26_] by the fit agreement (*R*_w_ value) shows that 2530 (18%) of the new clusters improve on the original fit, and 538 (4%) structures lowered the *R*_w_ value by 5% or more. The overview in Fig. S3 shows that, generally, clusters with ten or fewer Mo atoms give a significantly better fit to the data than larger clusters, and the average number of octahedra in all of the 538 best-fitting structures is 8.97. This number is more reasonable than the average from the α-[Mo_8_O_26_] fitting (4.5 Mo) due to the better fit to the PDF at high *r* shown in Fig. 6 ▸(*d*). The best-fitting cluster, containing nine Mo atoms, is shown in the inset in Fig. 6 ▸(*d*), along with its fit to the data. Notably, a dominating structural motif of the clusters originating from the β-[Mo_8_O_26_] cluster is three edge-sharing octahedra, often referred to as a triad, which form the main building block of many polyoxometalate clusters. In fact, 7333 of the 14 054 (52%) new β-[Mo_8_O_26_]-based structures contain a triad. However, if we look at structures improving the fits by 5% or more, 2062 of 2530 (82%) and 438 of 538 (81%) contain triad units. This significant increase in triad frequency reveals the triad to be an important structural motif in the supported molybdenum oxides, and this is confirmed by the same trend when the method is applied to a paratungstate cluster (Averbuch-Pouchot *et al.*, 1979 ▸) as the starting point (Table S5).

**Figure 7:**
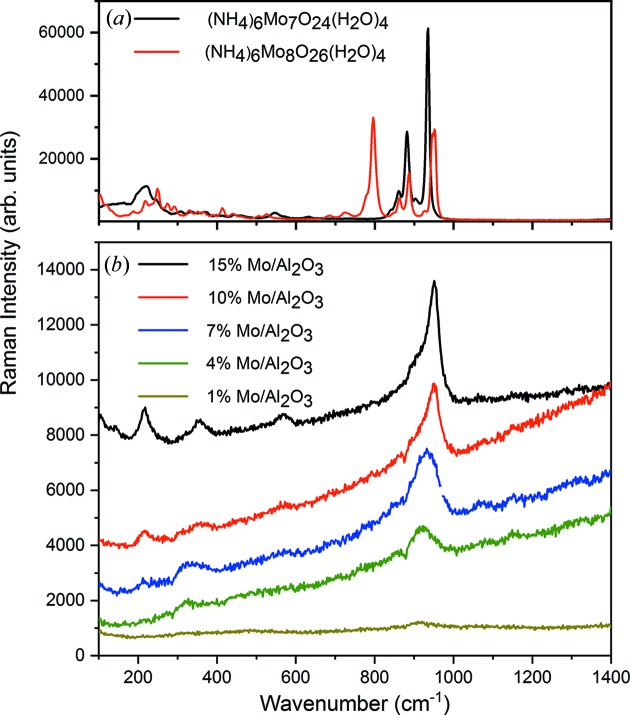
(*a*) Raman spectra of (NH_4_)_6_Mo_7_O_24_(H_2_O)_4_ and (NH_4_)_6_(Mo_8_O_26_)(H_2_O)_4_ in the solid state. (*b*) Micro-Raman spectra of MoO*_x_* supported on γ-Al_2_O_3_ nanoparticles with loadings from 1 to 15 wt%.

To gain further insight, the d-PDF analysis was complemented with Raman spectroscopy. The bands in the Raman spectra from the five samples [Fig. 7 ▸(*b*)] are all very broad when compared with the reference compounds, (NH_4_)_6_Mo_7_O_24_(H_2_O)_4_ and (NH_4_)_6_(Mo_8_O_26_)(H_2_O)_4_, whose spectra are seen in Fig. 7 ▸(*a*). This could be an indication of disorder as also observed in the d-PDFs. At low Mo loadings (1–7%), bands are visible at 920–950 cm^−1^ and *ca* 360 cm^−1^, which have previously been associated with Mo–O vibrational modes (Tian *et al.*, 2010 ▸; Mestl & Srinivasan, 1998 ▸). As the loading increases, bands at *ca* 220 and 560 cm^−1^ become more prevalent. These are known to be characteristic of Mo–O–Mo bridging (Tian *et al.*, 2010 ▸). The 220 cm^−1^ band and the 560 cm^−1^ band have been ascribed to an Mo–O–Mo bending mode and symmetric stretch, respectively, while the 360 cm^−1^ peak arises from an Mo–O bending mode (Tian *et al.*, 2010 ▸). Thus, the Raman analysis confirms the increasing polymerization of [MoO_6_] octahedra as the loading increases. The higher sensitivity of Raman spectroscopy also confirms the presence of molybdenum oxide for the sample with 1% Mo loading, which was difficult to resolve from the PDF analysis. A significant shift can be observed for the most prominent Mo–O band from *ca* 920 cm^−1^ in the 4% sample to 952 cm^−1^ in the 15% sample. Such a shift has previously been attributed to an increased degree of polymerization, which agrees well with the d-PDF observations (Cheng, 1979 ▸; Ng *et al.*, 1985 ▸). Note that other factors such as Mo—O bonding may also affect the Raman peak positions (Tian *et al.*, 2010 ▸), and from these highly disordered samples it is therefore difficult to extract quantitative structural information from the Raman data. The 920–950 cm^−1^ band narrows with increasing Mo content, indicating a higher structural order with higher Mo content.

The results from the PDF and Raman analyses show that there is not a single structural motif that can describe the experimental data of the nanostructured molybdenum oxide. However, it is clear from the refinements that the structures formed at high MoO_*x*_ loadings have motifs related to several known structures, but are not identical to the commonly known, monodisperse polyoxometalate clusters.

### 2.2. Molybdenum oxides supported on zeolites   

Having established the d-PDF method and automated cluster modelling for molybdenum oxides supported on γ-Al_2_O_3_ nanoparticles, we can now apply the same methodology to the zeolite-supported samples.

**Figure 8:**
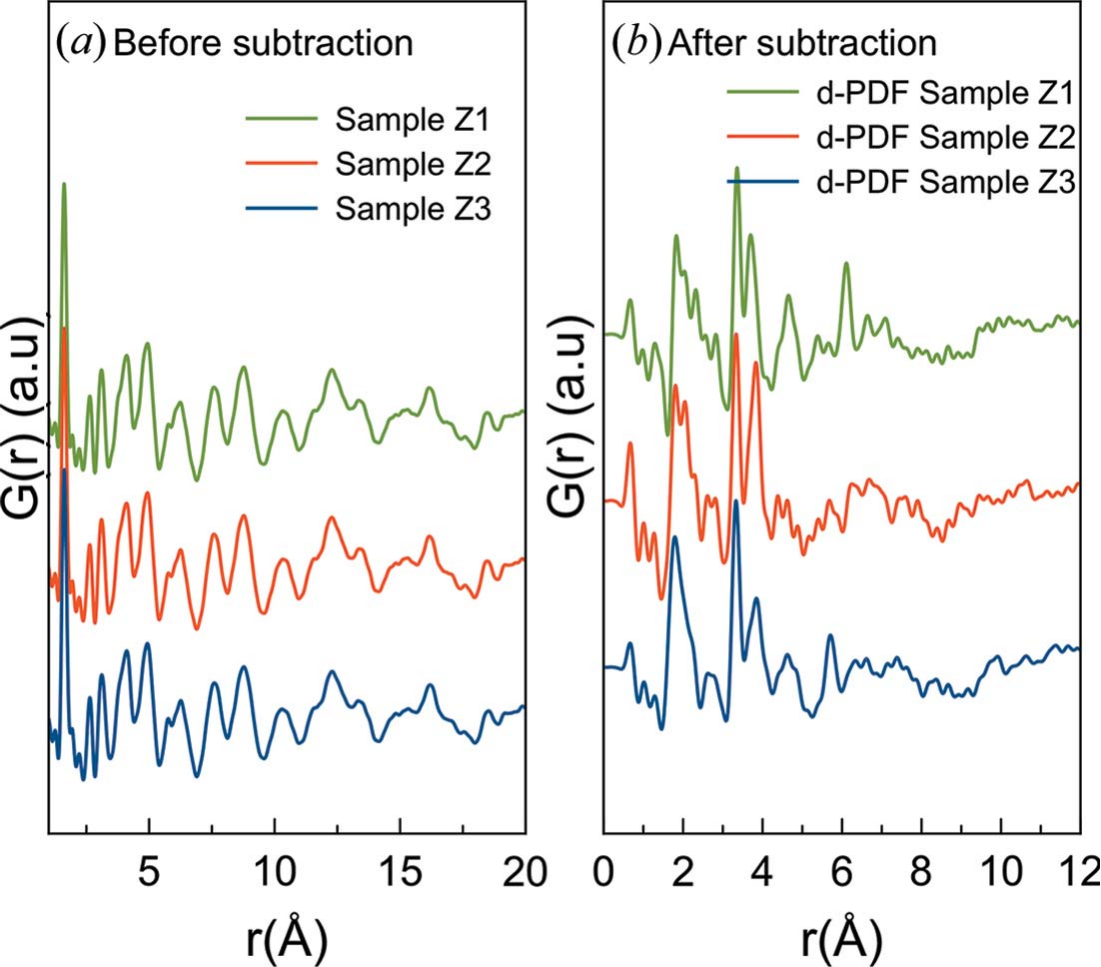
PDFs (*a*) and d-PDFs (*b*) obtained after subtraction of the pure zeolite signal for samples Z1, Z2 and Z3.

As discussed above, there is no prevailing structural model for MoO_*x*_ supported on zeolites (Li *et al.*, 2006 ▸). The optimum loading for catalysis is known to be *ca* 2–3 wt% molybdenum, and the molybdenum species supported on zeolites can form both in the channels of zeolites and on the surface. Depending on where the molybdenum oxide is situated, the structure is expected to differ (Ma *et al.*, 2000 ▸). On the external surface, it is suggested that the structure is similar to MoO_3_ and contains octahedral [MoO_6_] units (Ma *et al.*, 2000 ▸; Li *et al.*, 2006 ▸), while both isolated [MoO_*x*_] and more extended [Mo_5_O_12_] clusters have been suggested for the intra-zeolite structures (Gao *et al.*, 2015 ▸; Li *et al.*, 2006 ▸); however, the structures also depend on the zeolite nature (Okamoto *et al.*, 2002 ▸). Here, three zeolite-supported samples were analysed on similar zeolite (ZSM) supports and Mo loadings (3–4%): sample Z1 2.8% Mo on ZSM5-SAR50; sample Z2 3.0% Mo on ZSM5-SAR50; and sample Z3 4.2% Mo on ZSM5-SAR23. The PDFs of the MoO_*x*_-coated zeolites [Fig. 8 ▸(*a*)] are dominated by the zeolite signal and thus all appear similar. The corresponding *Q*-space data can be found in Fig. S5. The d-PDFs obtained by subtracting the PDF from the pure zeolite support from the data are shown in Fig. 8 ▸(*b*). From the first peak positions (1.8–2.5, 3.3 and 3.85 Å) it is clear that the MoO_*x*_ nanostructures supported on the zeolite samples are also built from edge- and corner-sharing motifs that are common to molybdenum oxides, and similar to the nanostructured MoO_*x*_ layer supported on γ-Al_2_O_3_ described above.

The d-PDFs show significant differences between the structures of the three samples. Firstly, the ratio of edge- and corner-sharing [MoO_*x*_] units differs in the three samples, as can be inferred by the change in relative intensity of the peaks at 3.3 and 3.8 Å. Furthermore, sample Z2 shows no clear structural peaks other than the first edge- and corner-sharing peaks, whereas both sample Z1 and sample Z3 show peaks beyond the first Mo–Mo coordination peaks, with clear peaks at 4.65 and 6.1 Å and at 4.65 and 5.71 Å, respectively.

**Figure 9:**
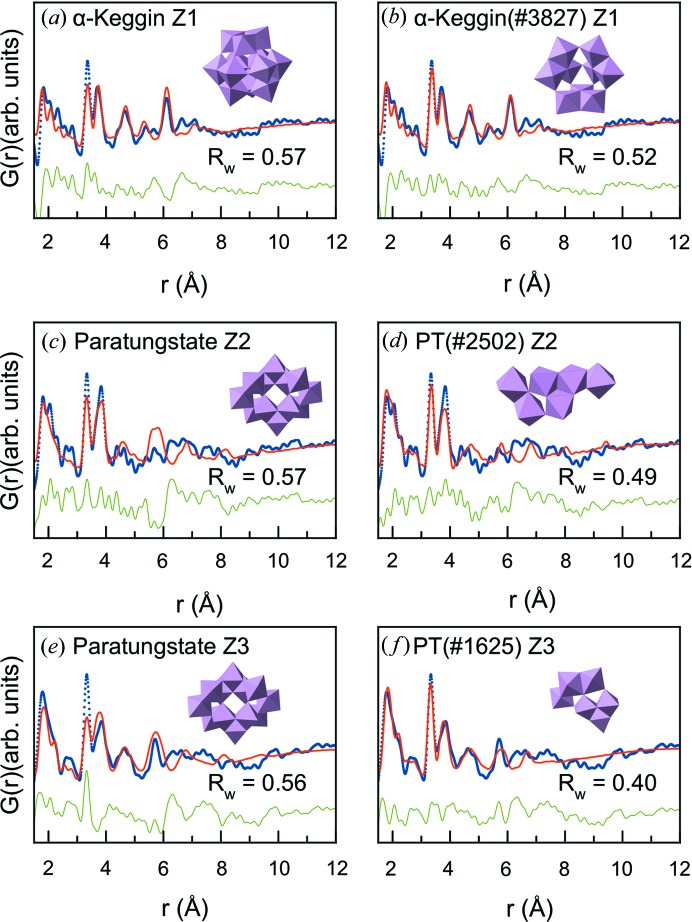
(*a*) Fit of the α-Keggin cluster structure to the d-PDF obtained from sample Z1. (*b*) Fit of the best-fitting α-Keggin-derived cluster structure to the d-PDF obtained from sample Z1. (*c*) Fit of the paratungstate cluster structure to the d-PDF obtained from sample Z2. (*d*) Fit of the best-fitting paratungstate-derived cluster structure to sample Z2. (*e*) Fit of the paratungstate cluster structure to the d-PDF obtained from sample Z3. (*f*) Fit of the best-fitting paratungstate-derived cluster structure to sample Z3. In all fits, the experimental PDF is displayed as blue dots, the calculated model as a red line and the difference curve as a green line.

The experimental d-PDFs were tested against the polyoxometalate cluster structures listed in Table S4, as also done for the γ-Al_2_O_3_-supported samples. Interestingly, an [Mo_12_O_40_] α-Keggin cluster (Boeyens *et al.*, 1976 ▸; Keggin, 1934 ▸) gave an excellent fit to the PDF of sample Z1 [Fig. 9 ▸(*a*)], while the paratungstate cluster described the main peaks in the PDF for sample Z2 and sample Z3, as seen in Figs. 9 ▸(*c*) and 9 ▸(*e*), respectively. The same algorithm as described above was used to determine the cluster fragments and thus structural motifs giving the best fit, with the results shown in Figs. 9 ▸(*b*), 9 ▸(*d*) and 9 ▸(*f*). Again, information about the sample structures is extracted by analysing the groups of clusters that give the best fit by using the *R*_w_ value as a metric. For sample Z1, 76 of the 2317 new, unique cluster structures tested yielded an improvement in *R*_w_ value by 5% or more. The cluster fragment giving the best fit [Fig. 9 ▸(*b*)] is very close to a full α-Keggin structure, just with one triad of octahedra missing, which improves the fit of the intensity ratio of the edge-sharing/corner-sharing peaks at 3.3 and 3.8 Å, and yields the best fit found using this method for any sample.

The PDFs for both samples Z2 and Z3 indicate that the samples contain smaller molybdenum oxide clusters than sample Z1. Firstly, for sample Z2, 91 of the 1944 (5%) new structures derived from the paratungstate cluster structure improved the fit by 5% or more. Common for these structures is that they result in weak PDF peaks above 4 Å, where the experimental data show very little structure. From this, we can infer that the main structural motifs present in the samples are small clusters of both edge- and corner-sharing molybdenum oxide units. The fit to sample Z3 improved significantly during the iterative search, as seen in Fig. 9 ▸(*f*). Of 1935 new structures 293 (15%) resulted in fit improvement by 5% or more, compared with the paratungstate starting model. Note that tests of the presence of [MoO_4_] tetrahedra in the structures were also performed on the zeolite-supported samples; however, the results were inconclusive. Because the Mo–Mo distances in *e.g.* edge-sharing octahedra and edge-sharing tetrahedra are indistinguishable, the structural motifs can only be identified from their extended Mo–Mo connection at higher *r* values. The MoO_*x*_ domains present in zeolites (especially sample Z2) appear too small to fully identify the molybdenum coordination. However, the best fits to the data were obtained using models containing octahedra, as presented in Fig. 9 ▸.

A common structural motif seen in the best-fitting cluster models in the zeolite samples is again the triad, consisting of three [MoO_6_] units that share edges, as depicted in the inset of Fig. S8. We quantify this again by looking at the occurrences of triads in the structures giving the best fit to the data. Considering the structures improving the fit *R*_w_ by 5% or more, 76 of 76 (100%) of the structures identified for sample Z1, 35 of 91 (38%, up from 15%) of the structures identified for sample Z2 and 127 of 180 (71%) of the structures identified for sample Z3 contain triads, again confirming the importance of the triad unit. In the PDF of all three samples, and especially samples Z2 and Z3, the corner-sharing peak at *ca* 3.8 Å is much broader that that originating from edge-sharing octahedra at 3.3 Å. For example, for sample Z3 the full width at half-maximum of the corner-sharing peak is approximately 70% larger than that of the edge-sharing peak, as seen from a single peak fitting in Fig. S7. From a structural point of view, this indicates a much broader distribution of corner-sharing distances. This points to the existence of a well-defined core consisting primarily of triads of edge-sharing [MoO_6_] octahedra, with these motifs more loosely connected by corner sharing. The broad distribution of distances reflects the larger degree of freedom in the connection, and the formation of a very disordered network, which is similar to what was observed for Mo/γ-Al_2_O_3_.

The size of a triad cluster is approximately 6.3 Å, when considering the distance between the two furthest oxygen atoms. Molecules of similar size have been shown to be able to reside in the ZSM5 zeolite network (Olson *et al.*, 1981 ▸), making it possible that the MoO_*x*_ structures observed here are situated both inside and on the surface of the zeolite. Further speculation can be made with respect to the coordination of the core cluster structures within the structure. As shown in Fig. S8 broad features appear in the d-PDF with a periodicity of approximately 12 Å in sample Z3. A possible explanation for the periodicity in the structure is a packing of the core clusters in the large pores of the zeolite, which are spaced *ca* 12 Å apart, as indicated in Fig. S8. However, other structural effects, *e.g.* the presence of oligomeric chains with cores spaced 12 Å apart, could result in similar features in the PDF, and the effect somewhat resembles that seen in C_60_ bucky balls (Juhás *et al.*, 2006 ▸) and small organic compounds (Prill *et al.*, 2015 ▸) with well-defined intramolecular peaks and broad intermolecular peaks.

Raman spectra were also collected for the zeolite-supported MoO_*x*_ samples and can be found in Fig. S9. The samples show higher heterogeneity of the MoO_*x*_ presence on the support and less signal from the MoO_*x*_ layer, due to a poorer distribution on the larger zeolite particles compared with the MoO_*x*_/γ-Al_2_O_3_ samples. The limited conclusion that can be drawn from these data is that the Mo–O bands between 920 and 950 cm^−1^ can be observed, similar to the MoO_*x*_/γ-Al_2_O_3_ samples, but the Mo–O–Mo bridging bands are harder to observe.

## 3. Conclusions   

Using differential pair distribution function analysis and automated cluster fitting, we have shown that the atomic structure of nanoscale MoO_*x*_ supported on γ-Al_2_O_3_ nanoparticles can be described by a distribution of polymeric [MoO_*x*_] cluster fragments closely related to known polyoxometalate structures. The structures present on the surface are not monodisperse, well-defined polyoxometalates, but the structural motifs are very reminiscent of those known from polyoxometalate clusters. Notably, triads built from [MoO_6_] have been identified as an important structural motif. When supported on ZSM5 zeolites, the MoO_*x*_ structures share many similarities with those observed on γ-Al_2_O_3_, however with much smaller structural coherence lengths. Both Keggin-like and paratungstate-like clusters have been identified in the zeolite-supported samples, which are small enough to fit in the cavities in the zeolite structure. All cluster structures have been identified by a new approach to structural characterization and identification, taking advantage of known, stable cluster structures and polyoxometalate chemistry. By using known, chemically sensible structures as a starting point, we automatically generate a large number of new, related structures that both provide better fits and allow us to extract information on average structural motifs present in the sample. The method has the potential to become a valuable tool in handling the challenges of characterizing nano­structures that are often both highly polydisperse and disordered.

## 4. Experimental   

### 4.1. Material preparation   

The Mo/γ-Al_2_O_3_ samples were prepared by flame spray pyrolysis, as described in detail elsewhere (Høj *et al.*, 2013 ▸, 2014 ▸). In brief, solutions of molybdenum 2-ethyl hexanoate and aluminium acetylacetonate in toluene were sprayed with oxygen as dispersion gas into a premixed methane/oxygen flame. The flammable mixtures combusted completely with entrainment of air from the surroundings and solid oxides condensed from the gas phase. Due to the difference in boiling point the alumina condenses first. The solid products were collected on glass fibre filters.

The Mo/zeolite samples were synthesized via a continuous hydrothermal flow method in a purpose-built reactor (Ma *et al.*, 2000 ▸; Kallesøe *et al.*, 2014 ▸). The zeolite is mixed into an Mo^VI^ precursor solution made from molybdenum acetylacetonate (99.9%, Sigma Aldrich), polyvinylpyrrolidine (99.99%, Sigma Aldrich), absolute ethanol (99.9%) and ethylene glycol (99.9%, Alfa Aesar). As the suspension is pumped through the reactor, the precursor (Mo + zeolite) meets the solvent (H_2_O) which is heated to 723 K and at a pressure of 210 bar (1 bar = 100 kPa). At the high temperature and pressure, the Mo^VI^ precursor is reduced and hydrolysed in a fast reaction forming molybdenum oxide in the zeolite network. The product of the hydrothermal synthesis was then washed in ethanol to remove excess solvent and organic residue, dried for 24 h and calcined in air at 773 K for 6 h.

### 4.2. PDF   

X-ray total scattering data were obtained at the Advanced Photon Source at Argonne National Laboratory at beamline 11-ID-B. The powders were packed in Kapton capillaries. The X-ray wavelength was 0.2112 Å with a detector distance of 150 mm in the RA-PDF setup (Chupas *et al.*, 2003 ▸). The obtained data were integrated using *Dioptas* (Prescher & Prakapenka, 2015 ▸) and Fourier transformed to obtain the PDF using *xPDFsuite* (Yang *et al.*, 2015 ▸) with the following parameters: *Q*_min_ = 0.5 Å^−1^, *Q*_max_ = 24 Å^−1^, *Q*_maxins_ = 24 Å^−1^ and *R*_poly_ = 0.9. d-PDFs were obtained by subtracting the normalized PDFs of the support from those of the samples, as described further in the main text. The PDFs were modelled using *PDFgui* (Farrow *et al.*, 2007 ▸) and *Diffpy-CMI* (Juhás *et al.*, 2015 ▸). For all refinements, the instrumental PDF damping was included through the Qdamp parameter, which was determined through refinement of the PDF obtained from a bulk CeO_2_ standard, measured in the same instrumental configuration.

### 4.3. Micro-Raman spectroscopy   

Raman measurements were performed using a micro-Raman setup in backscattering geometry. The 514.5 nm line of an argon-ion laser (CVI Melles-Griot 35MAP431-200) was used (430 µW for the γ-Al_2_O_3_ samples and 125 µW for the zeolite samples, above the objective). The beam was focused in an inverted confocal microscope (Olympus IX71) by an Olympus 100X, 1.4-NA oil immersion objective into a diffraction-limited spot. Raman spectra were collected using a Princeton Instruments SPEC 10:100 B/LN-eXcelon CCD detector and an SP 2356 spectrometer with a 600 grooves per millimetre grating. An LL01-514 filter (Semrock) was used to clean the laser light, a 30:70 beam splitter (XF122 Omega Optical) was used instead of a dichroic mirror and two LP02-514RE filters (Semrock) were used to block the remaining laser light in the detection path. *X*-axis calibration was performed with a neon spectral lamp (6032 Newport). No *Y*-axis corrections or background removal procedures were performed. Only a constant value was added or subtracted for display purposes. The spectra were not averaged. Because a limited amount of material is probed in the confocal micro-Raman experiments, heterogeneities in the Raman spectra might be present. The signal comes from a diffraction-limited area, and the signal-to-noise ratio depends on the amount of sample present, which can vary from spot to spot. Three or four spectra were recorded for each sample and all the spectra can be found in Figs. S9 and S10.

## 5. Related literature   

The following reference is cited in the supporting information: Kihlborg (1963 ▸).

## Supplementary Material

Details on models and modelling results; Raman data. DOI: 10.1107/S1600576719016832/kc5101sup1.pdf
