# Supplementary material for: Structure analysis of supported disordered molybdenum oxides using pair distribution function analysis and automated cluster modelling
Source: J Appl Crystallogr. 2020 Feb 1;53(Pt 1):148–58. doi: 10.1107/S1600576719016832 (PMC6998784; doi:10.1107/S1600576719016832)
Supplement: Supplementary file 1 [file j-53-00148-sup1.pdf]

JOURNAL OF
APPLIED
CRYSTALLOGRAPHY

Volume 53 (2020)

Supporting information for article:

Structure analysis of supported disordered molybdenum oxides using pair distribution function analysis and automated cluster modelling

Troels Lindahl Christiansen, Emil T. S. Kjær, Anton Kovyakh, Morten L. Röderen, Martin Høj, Tom Vosch and Kirsten M. Ø. Jensen

Refinement results for real space Rietveld fits of Mo/Al_2O_3 samples

Table S1: Results from Real space Rietveld refinements of the PDFs obtained from Mo/Al_2O_3 samples prior to the calculation of the d-PDFs. In the long-range (10 Å – 60 Å) the tetragonal γ-Al_2O_3 developed by Paglia et al(Paglia *et al.*, 2003) was used, while the fine-scale nanostructure γ-Al_2O_3 model was applied in the local range (1.5 Å – 8 Å).(Paglia *et al.*, 2006)

	a (Å)	b (Å)	b (Å)	Al_Biso ($Å^2$)	O_Biso ($Å^2$)	SPdiameter (Å)
1% Mo/Al_2O_3 (10 Å – 60 Å)	5.620	5.620	7.829	0.015	0.033	84.461
4% Mo/Al_2O_3 (10 Å – 60 Å)	5.619	5.619	7.833	0.015	0.033	74.864
7% Mo/Al_2O_3 (10 Å – 60 Å)	5.618	5.618	7.835	0.015	0.034	73.667
10% Mo/Al_2O_3 (10 Å – 60 Å)	5.616	5.616	7.834	0.015	0.034	85.338
15% Mo/Al_2O_3 (10 Å – 60 Å)	5.615	5.615	7.845	0.015	0.034	81.537
γ-Al_2O_3 (10 Å – 60 Å)	5.618	5.618	7.832	0.015	0.032	77.171
1% Mo/Al_2O_3 (1.5 Å – 8 Å)	3.405	2.789	7.074	0.017	0.013	----
4% Mo/Al_2O_3 (1.5 Å – 8 Å)	3.406	2.789	7.080	0.018	0.014	----
7% Mo/Al_2O_3 (1.5 Å – 8 Å)	3.404	2.789	7.083	0.018	0.013	----
10% Mo/Al_2O_3 (1.5 Å – 8 Å)	3.402	2.789	7.085	0.016	0.014	----
15% Mo/Al_2O_3 (1.5 Å – 8 Å)	3.391	2.794	7.086	0.014	0.013	----
γ-Al_2O_3 (1.5 Å – 8 Å)	3.405	2.788	7.076	0.017	0.013	----

Refinement results for pseudo crystalline fits of the 15% Mo/Al2O3 d-PDF

Table S2: Refinement results for pseudo crystalline fits of the 15% Mo/Al_2O_3 d-PDF. A Pbnm model by Kihlborg (Kihlborg, 1963) was used to fit MoO_3, while the heptamolybdate and octamolybdate models were derived from $(NH_4)_6Mo_7O_{24}(H_2O)_4$ (Evans *et al.*, 1975) and $(NH_4)_6(Mo_8O_{27})(H_2O)_4$, (Boschen *et al.*, 1974) respectively, by removing all atoms except Mo atoms and O atoms bonded to Mo from the unit cell. The space groups used were P 1 2_1/c and P 1 2_1/n for heptamolybdate and octamolybdate, respectively.

	a (Å)	b (Å)	c (Å)	Beta (°)	Mo Biso (Å^2)	O Biso (Å^2)	SPdiameter (Å)
MoO_3	3.92123	13.4607	3.73917	-	0.0018	0.14	5.22
Heptamolybdate	12.9722	10.1991	16.665	88.8905	0.0001	0.0019	20.0
Octamolybdate	9.73237	10.867	15.0132	110.448	0.00022	0.0021	10.4418

Refinement results for 15% Mo/Al_2O_3 d-PDF and Mo/Zeolite d-PDFs using cluster structural models

Table S3: Refinement results for 15% Mo/Al_2O_3 d-PDF and Mo/Zeolite sample d-PDFs with finising cluster models. The table shows values from both the starting clusters and the derived clusters generated by cluster iteration. The Delta2 value has been fixed to 5 for all fits.

	Zoom x	Zoom y	Zoom z	Mo Biso (Å^2)	O Biso (Å^2)	Scale
β-octamolybdate (15% Mo/Al2O3)	0.982	1.003	0.980	0.429	0.953	0.147
β-octamolybdate permutation #6009 (15% Mo/Al2O3)	1.000	1.006	0.9863	0.2338	1.077	0.190
α-octamolybdate	0.982	0.996	1.005	0.389	0.300	0.116
α-octamolybdate permutation #252	0.980	0.980	1.08	0.300	1.08	0.160
α-Keggin (sample Z1)	1.020	1.002	0.9852	0.3919	1.781	0.039
Paratungstate (sample Z2)	0.981	1.020	1.008	0.767	0.296	0.088
Paratungstate (sample Z3)	0.980	Z.001	1.016	0.814	2.670	0.169
α-Keggin Perm. #3827 (sample Z1)	1.007	0.981	1.020	0.282	0.971	0.039
PT Perm #2502 (sample Z2)	0.980	1.016	1.001	0.425	1.347	1.065
PT Perm 1625 (sample Z3)	1.000	0.984	1.020	0.556	2.651	0.193

Calculating d-PDFs from Q-space subtraction

In the main paper, we demonstrated that d-PDFs could be generated by subtracting the PDF of the supported molybdenum oxide samples with the PDF of the support nanoparticles. In this process, the PDFs obtained from the support are scaled so that the intensity matches in the region where only PDF peaks from the support material are present.

It is also possible to do the subtraction in Q-space, i.e. before the Fourier transform done to obtain the PDFs. Figure S1A shows the measured total scattering data of the sample (15% Mo) and the support. The green curve is the difference signal, which is used in the Fourier transform. Small Bragg peaks can be seen in the difference curve which can be assigned to the support and their presence is a consequence of imperfect support subtraction. Figure S1B shows the PDF generated via Q-space subtraction compared to the one generated by r-space subtraction. The two PDFs are essentially identical, and from the difference curve it appears that the difference is a question of scaling of the PDFs. Thus, the two methods of calculating the PDFs are both viable. However, the method of subtracting in r-space is preferred due to the easy and reliable method of scaling the PDF from the support to the correct intensity.

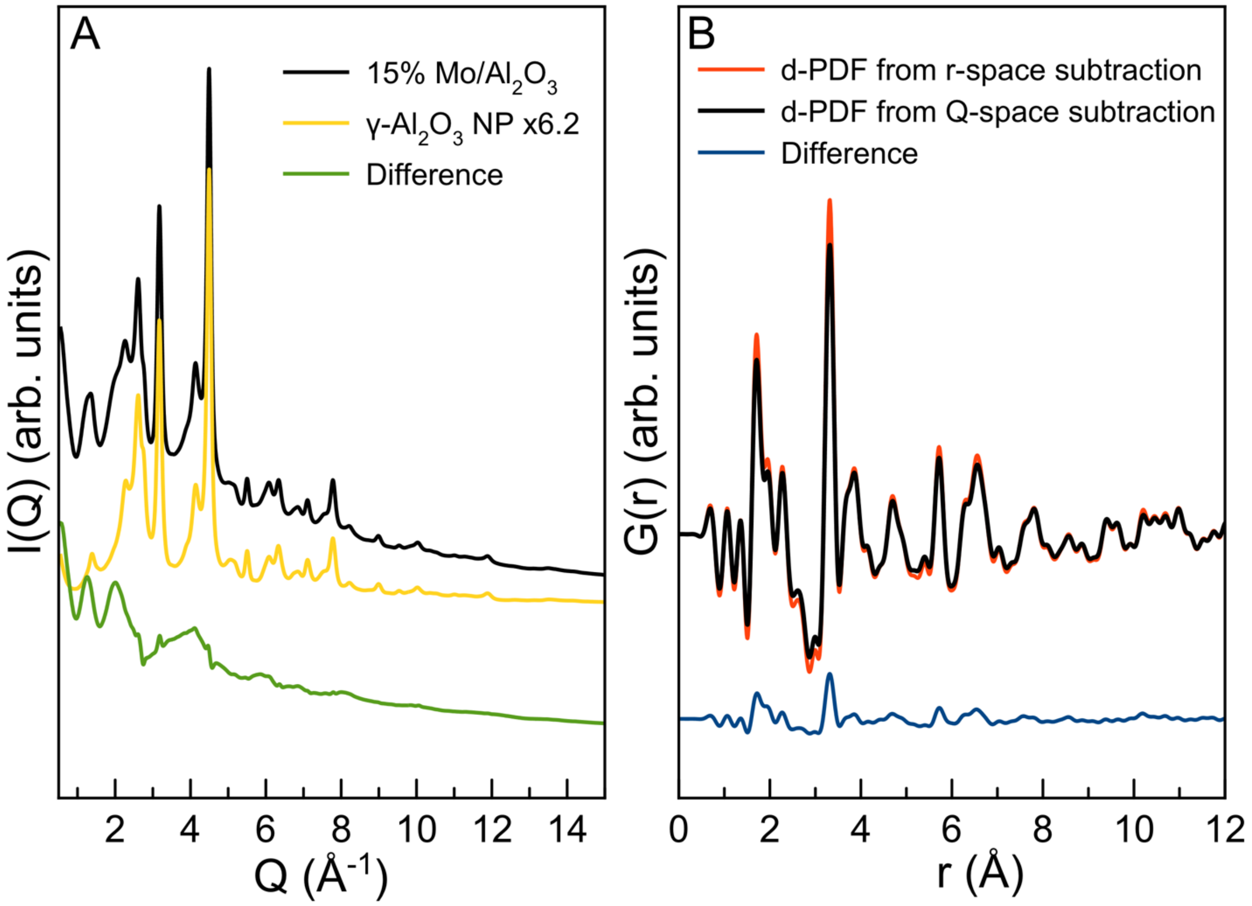

Figure S1: Comparison of calculating PDFs in Q-space vs. r-space. A: Raw total scattering data and the subtraction of the two. B: comparison of d-PDFs from the two methods.

Fits of PDFs from heptamolybdate and tungsten Kegging clusters in solution

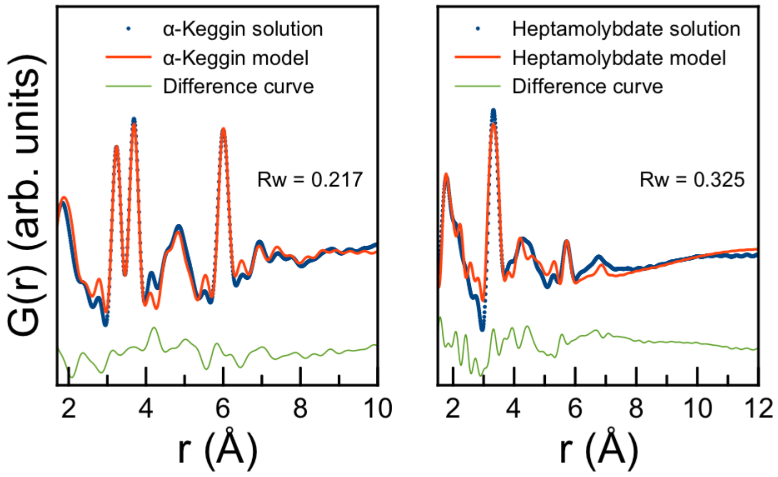

Figure S2: PDFs of monodisperse cluster in solution of left: 0.05 M ammonium metatungstate in H_2O, and right: 0.14 M ammonium heptamolybdate in H_2O. The refinement of the ammonium metatungstate uses an α-Keggin model and has been described elsewhere,(Juelsholt *et al.*, 2019) while the ammonium heptamolybdate model was taken from $(NH_4)_6Mo_7O_{24}(H_2O)_4$ *(Evans et al., 1975)*

List of molybdenum and tungsten polyoxometalate clusters for automated cluster modelling

Table S4: List of clusters evaluated when considering cluster to use for the iterative cluster search. For the clusters usually known from tungsten polyoxometalate chemistry, W was replaced with Mo in the model.

	Number of Mo atoms in structure
Heptamolybdate	7
α-octamolybdate	8
β-octamolybdate	8
β-octamolybdate (2 units)	16
α-Keggin	12
β-Keggin	12
γ-Keggin	12
δ-Keggin	12
ε-Keggin	12
Paratungstate	12
Tungstate Y	12
Decamolybdate	10
Octadecamolybdate	18

Overview of R-values in automated cluster modelling

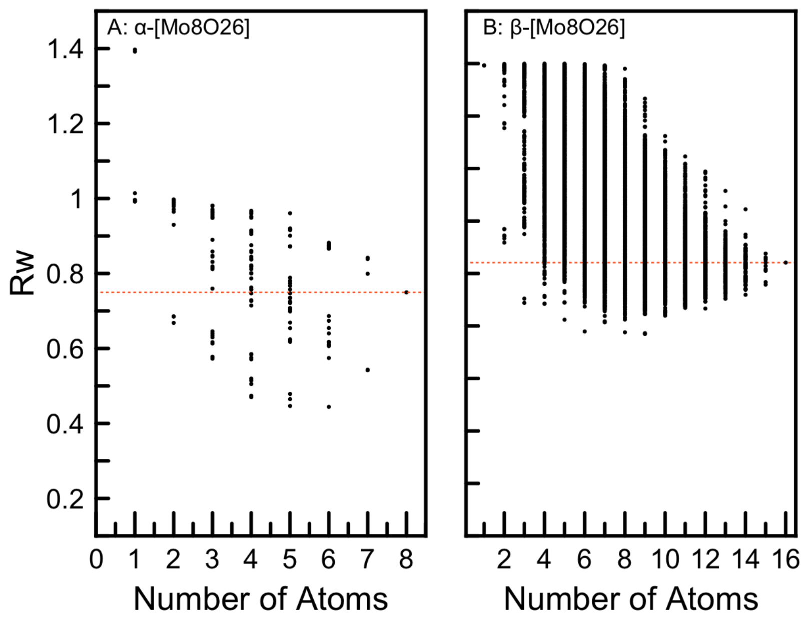

Figure S3: Rw-values from fits of structures derived from A: α-octamolybdate and B: β-octamolybdate, plotted as a function of number of Mo atoms in the new cluster structure. All fits were done in the 3-12 Å range. The dotted lines indicates the R_w value obtained for the starting clusters.

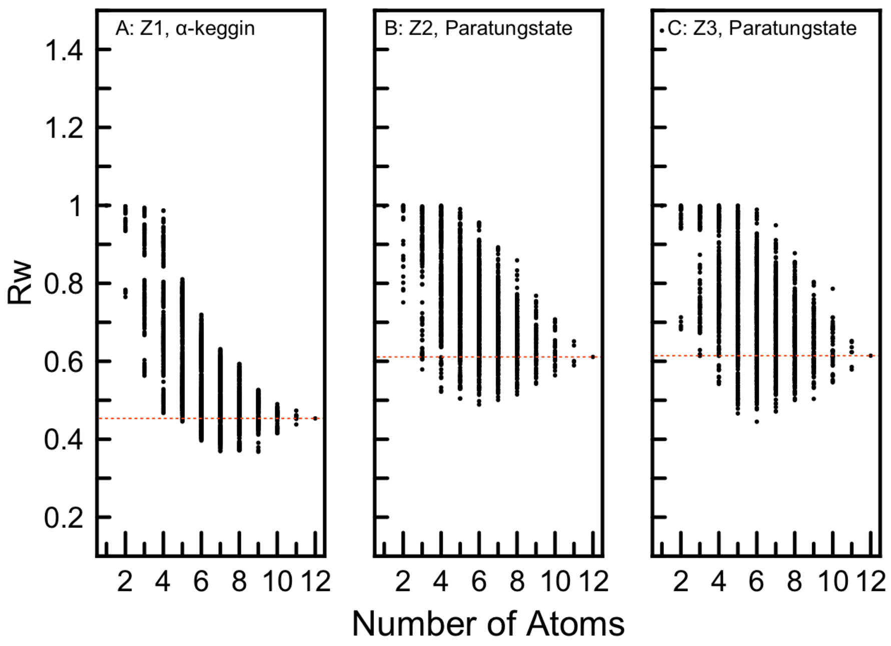

Figure S4: R-values from fits of Z1,Z2 and Z3 using structures derived from α-Keggin or paratungstate. All fits were done in the 3-12 Å range. The dotted lines indicates the R_w value obtained for the starting clusters.

Raw total scattering data from MoO_X coated zeolites

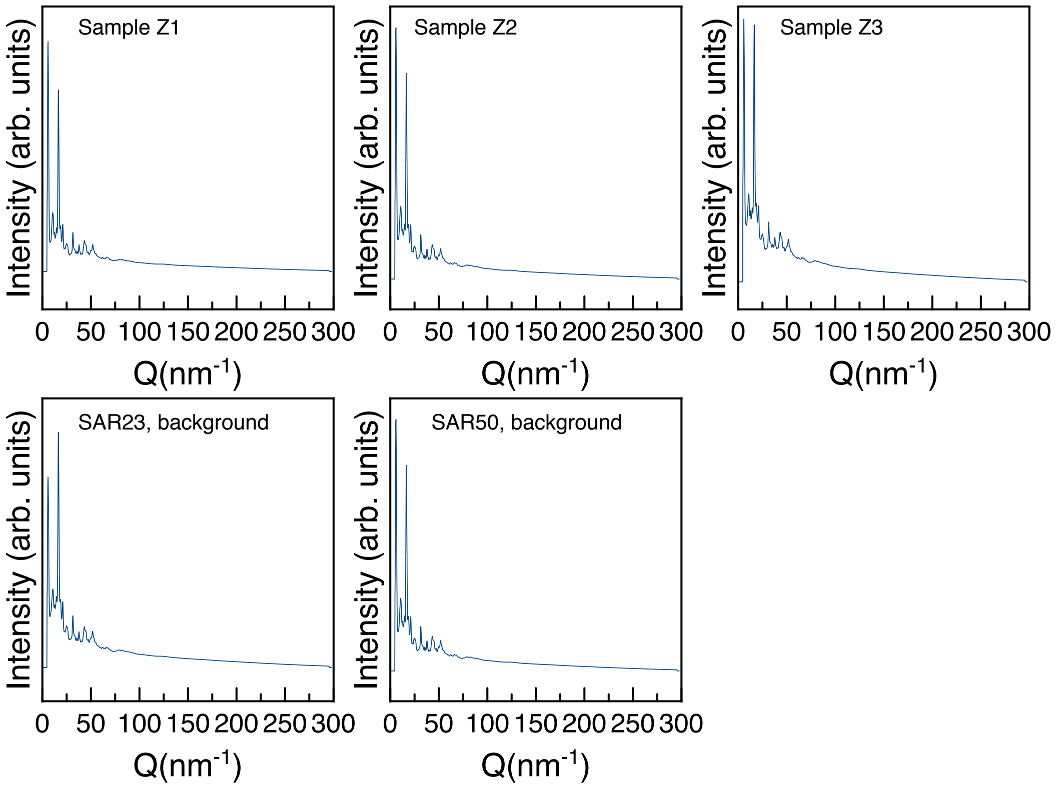

Figure S5: X-ray total scattering data used for calculation of PDFs for sample Z1, Z2, and Z3 along with background measurements of the pure zeolites.

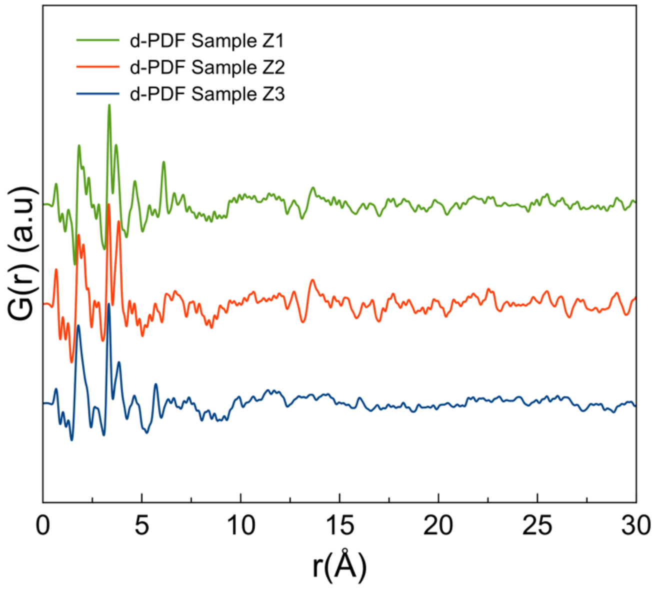

Figure S6: full range of PDFs calculated for sample Z1, Z2, and Z3.

Results from analysis of permutation models

Table S5: Main results extracted from the automated data modelling.

Sample	Starting model	#models	#unique models	#number of improved models unique	#number of unique models improved by 5% or more	#Triads in all unique model	#Triads in improved models	#Triads in improved models by 5% or more	Best Rw values (3 – 12 Å)
15% Mo/Al_2O_3	Octamolybdate	65536	14054	2530	538	7333 (52 %)	2062 (82 %)	438 (81%)	0.4847
15% Mo/Al_2O_3	Paratungstate	4096	2210	602	311	400 (18%)	271 (45%)	183 (58%)	0.5116
Z1	α-Keggin	4096	2317	330	76	937 (40%)	322 (98%)	76 (100%)	0.3679
Z2	Paratungstate	4096	1944	304	91	298 (15%)	105 (35%)	35 (38%)	0.4890
Z3	Paratungstate	4096	1935	390	180	293 (15%)	210 (54%)	127 (71%)	0.4450

Counting number of unique structures

To count the number of unique structures we compare the Rw-values obtained when the model is fitted to the data. We assume that identical models have the same number of Mo and O atoms and yield the same Rw-value. Identical models occur due to symmetry in the starting models, however while the starting clusters appear symmetrical when considering the octahedra/tetrahedras in the starting model, the individual octahedras are distorted in the Mo – O connection, which breaks the symmetry. Thus, the Rw-values are only identical if we round them to take into account the small differences in the octahedras. If we round on the third decimal, we see for the permutations of paratungstate on Z2 and Z3 that 1944 and 1935 unique structures are found, respectively. The small deviation between these two numbers indicate that the method, while not perfect, is a valid way of identified identical structures.

Permutation investigations using polyoxometalates with tetrahedra

Table S 6: some results extracted from through the permutation method for polyoxometalates containing tetrahedral $[MoO_4]$ units.

Sample	Starting model	#models	#unique models	#number of improved models unique	#number of unique models improved by 5% or more	#Tetrahedras in all unique model	#Tetrahedras in improved models	#Tetrahedras in improved models by 5% or more	Best Rw values (3 – 12 Å)
15% Mo/Al_2O_3	α-Mo_8O_{26}	256	160	57	45	120 (75 %)	30 (53%)	18 (40 %)	0.4442
15% Mo/Al_2O_3	Decamolybdate	1024	641	137	60	479 (75%)	89 (65 %)	35 (58 %)	0.4636
Z1	α-Mo_8O_{26}	256	145	42	13	106 (73%)	26 (62 %)	13 (100%)	0.7177
Z1	Decamolybdate	1024	570	122	7	428 (75 %)	73 (65 %)	5 (71 %)	0.7224
Z2	α-Mo_8O_{26}	256	142	14	0	107 (75 %)	14 (100%)	0 (0%)	0.4933
Z2	Decamolybdate	1024	612	78	26	453 (74 %)	58 (74 %)	20 (77 %)	0.5313
Z3	α-Mo_8O_{26}	256	159	47	32	125 (79 %)	30 (64 %)	22 (69 %)	0.4710
Z3	Decamolybdate	1024	591	26	3	438 (74%)	20 (77 %)	3 (100 %)	0.4561

Gaussian peak fitting of edge- and corner-sharing peaks in Mo/zeolite d-PDFs

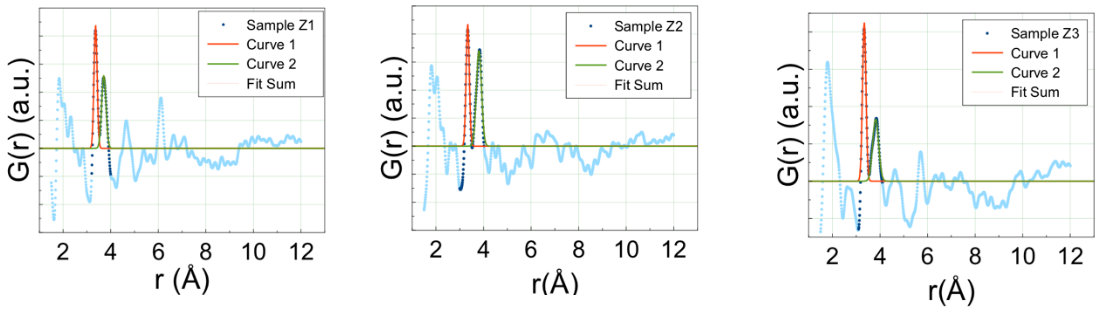

Figure S7: Gaussian peak fitting of the Mo-Mo peaks in samples Z1, Z2 and Z3. The Half-width Half-max parameters obtain were as follows: Sample Z1: Curve 1_{HWHM} = 0.0779, Curve 2_{HWHM} = 0.1072; Sample Z2: Curve 1_{HWHM} = 0.0792 Curve 2_{HWHM} = 0.1270; Sample Z3: Curve 1_{HWHM} = 0.0852 Curve 2_{HWHM} = 0.1474

Periodicity in zeolite d-PDFs

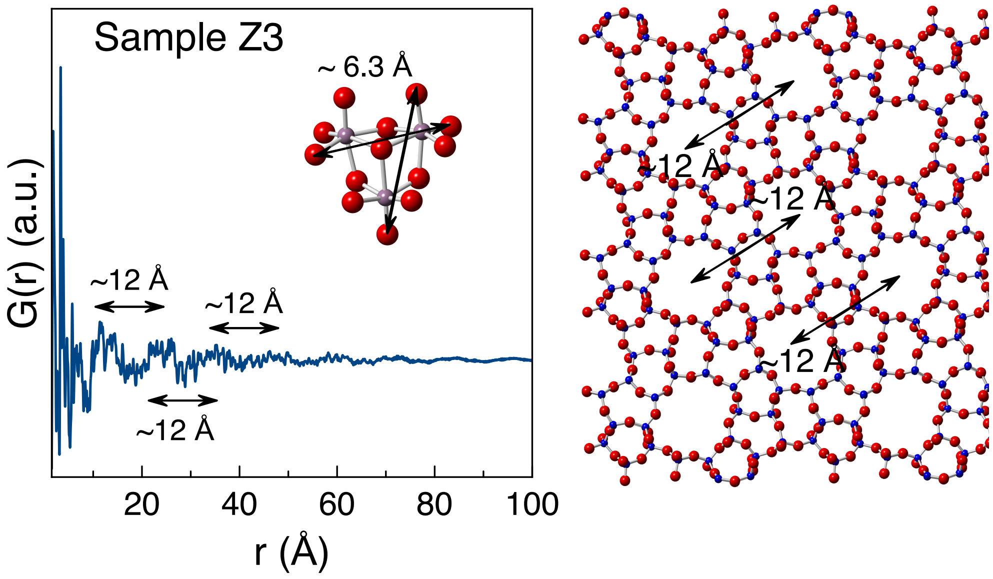

Figure S8: Demonstration of periodicity in the d-PDF of sample Z3. The periodicity of 12 Å fits with distance between pores in the zeolite. The insert also shows the size of the triad unit obtained from cluster iterations.

Raman spectra

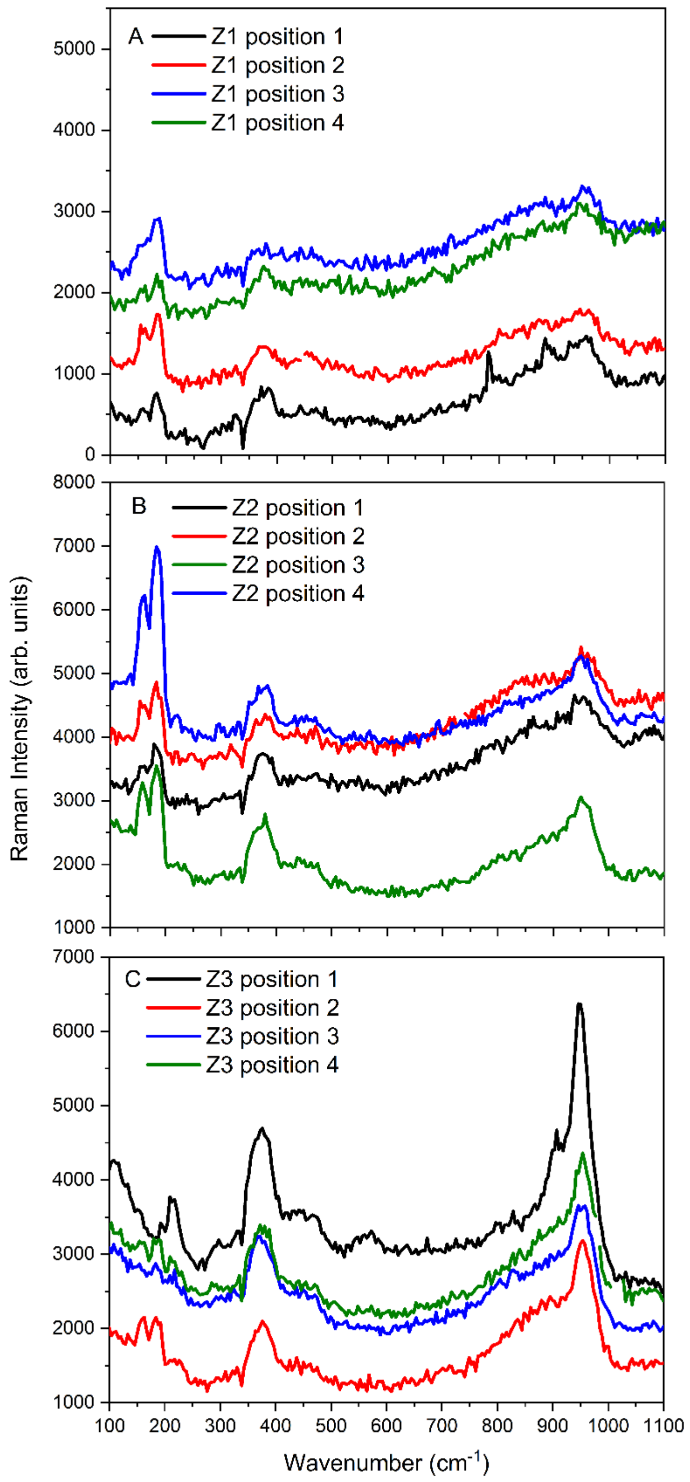

Figure S9: Micro-Raman spectra at four different positions in the sample. A) zeolite sample Z1. B) zeolite sample Z2. C) zeolite sample Z3.

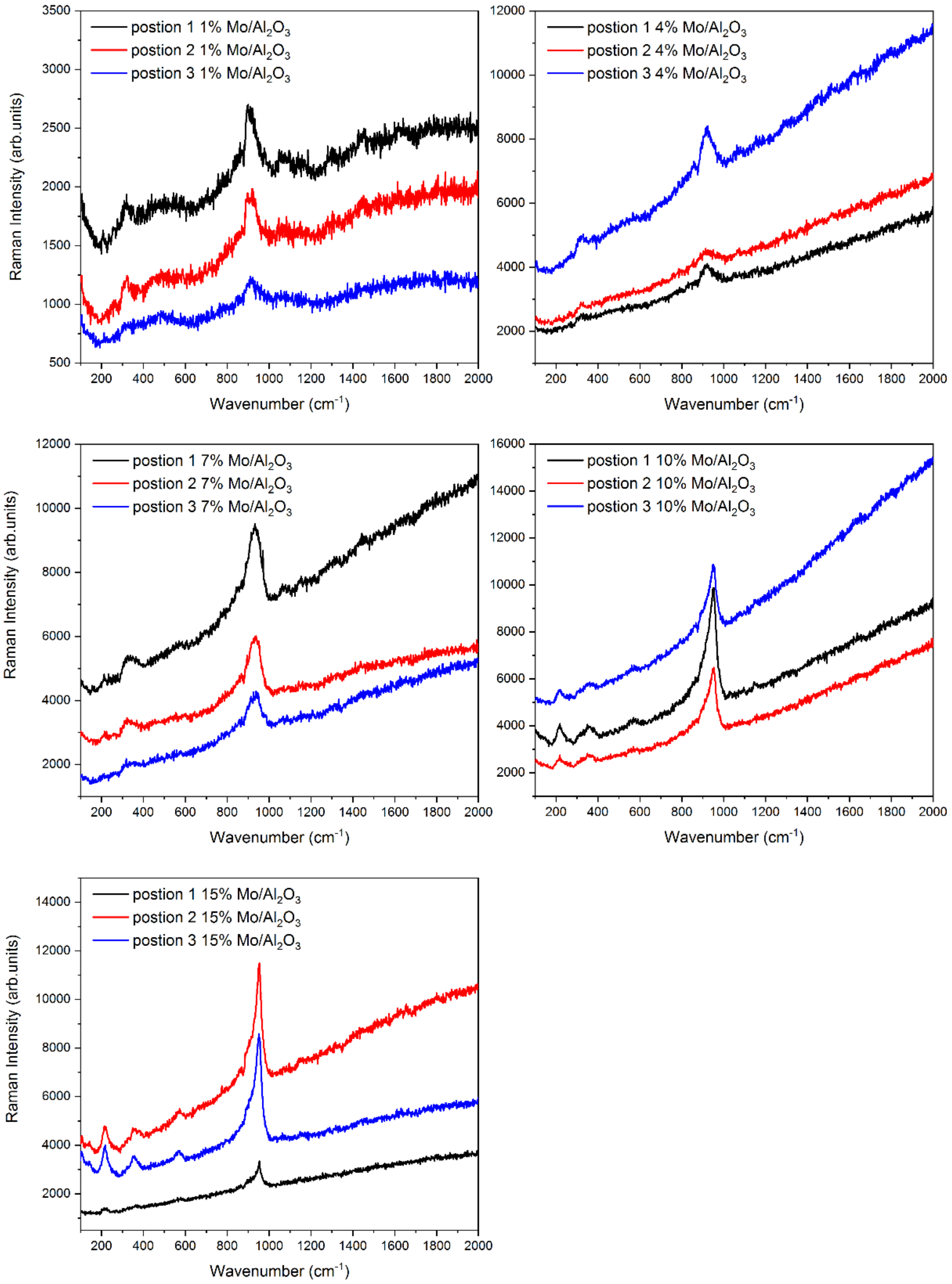

Figure S10: Micro-Raman spectra at three different positions in the samples with MoOx with different loading on γ-Al2O3 nanoparticles.

References:

Boschen, I., Buss, B. & Krebs, B. (1974). *Acta Crystallogr., Sect. B* **30**, 48-56.

Evans, H. T., Gatehouse, B. M. & Leverett, P. (1975). *Dalton Trans.* 505-514.

Juelsholt, M., Lindahl Christiansen, T. & Jensen, K. M. Ø. (2019). *J. Phys. Chem. C* **123**, 5110-5119.

Kihlborg, L. (1963). *Arkiv foer Kemi* **21**, 357 - 364.

Paglia, G., Božin, E. S. & Billinge, S. J. L. (2006). *Chem. Mater.* **18**, 3242-3248.

Paglia, G., Buckley, C. E., Rohl, A. L., Hunter, B. A., Hart, R. D., Hanna, J. V. & Byrne, L. T. (2003). *Phys. Rev. B: Condens. Matter* **68**, 144110.
